# Plasma concentrations of neurofilament light, p-Tau231 and glial fibrillary acidic protein are elevated in patients with chronic kidney disease and correlate with measured glomerular filtration rate

**DOI:** 10.1186/s12882-025-04130-2

**Published:** 2025-05-09

**Authors:** Torunn Axelsson, Henrik Zetterberg, Kaj Blennow, Burak Arslan, Nicholas J. Ashton, Markus Axelsson, Maria K. Svensson, Aso Saeed, Gregor Guron

**Affiliations:** 1 Department of Molecular and Clinical Medicine/Nephrology, Institute of Medicine, The Sahlgrenska Academy at the University of Gothenburg, Gothenburg, Sweden; 2 Department of Psychiatry and Neurochemistry, Institute of Neuroscience and Physiology, The Sahlgrenska Academy at the University of Gothenburg, Mölndal, Sweden; 3 Clinical Neurochemistry Laboratory, Sahlgrenska University Hospital, Mölndal, Sweden; 4 Department of Neurodegenerative Diseases, UCL Institute of Neurology, Queen Square, London, UK; 5 UK Dementia Research Institute at UCL, London, UK; 6 Hong Kong Center for Neurodegenerative Diseases, Clear Water Bay, Hong Kong China; 7 Wisconsin Alzheimer’s Disease Research Center, University of Wisconsin School of Medicine and Public Health, University of Wisconsin-Madison, Madison, WI USA; 8 Paris Brain Institute, ICM, Pitié-Salpêtrière Hospital, Sorbonne University, Paris, France; 9 Neurodegenerative Disorder Research Center, Division of Life Sciences and Medicine, and Department of Neurology, Institute on Aging and Brain Disorders, University of Science and Technology of China and First Affiliated Hospital of USTC, Hefei, P.R. China; 10 Department of Clinical Neuroscience, Institute of Neuroscience and Physiology, The Sahlgrenska Academy at the University of Gothenburg, Gothenburg, Sweden; 11 Department of Medical Sciences, Renal Medicine, Uppsala University, Uppsala, Sweden; 12 Uppsala Clinical Research Centre, Uppsala, Sweden

**Keywords:** Chronic kidney disease, Glial fibrillary acidic protein, Glomerular filtration rate, Neurofilament light, Tau

## Abstract

**Background:**

Patients with chronic kidney disease (CKD) have a high prevalence of cerebrovascular disease and cognitive impairment. The objective was to analyse whether plasma concentrations of neurofilament light chain (NfL), glial fibrillary acidic protein (GFAP) and phosphorylated Tau231 (p-Tau231) are elevated in patients with CKD and to identify independent predictors of these biomarkers, with an emphasis on the role of measured glomerular filtration rate (mGFR).

**Methods:**

In this cross-sectional cohort study, we included 110 patients with CKD stages 3 and 4 (estimated GFR 15–59 ml/min/1.73 m^2^) without manifest cerebrovascular disease or dementia, and 55 healthy controls. Biomarkers of neurological disorders were measured with ultrasensitive single molecule array methods.

**Results:**

Plasma concentrations (median [IQR]) of NfL (37.5 [22.1–47.5] vs. 13.4 [10.5–16.7] ng/L, *p* < 0.001), p-Tau231 (25.7 [19.1–38.7] vs. 13.9 [10.5–16.3] ng/L, *p* < 0.001) and GFAP (190 [140–281] vs. 153 [116–211] ng/L, *p* < 0.001) were elevated in patients with CKD vs. controls. Measured GFR was negatively correlated with NfL (*r* = − 0.706, *p* < 0.001), p-Tau231 (*r* = − 0.561, *p* < 0.001), and GFAP (*r* = − 0.385, *p* < 0.001). In multivariable linear regression models, mGFR was an independent predictor of log-transformed plasma concentrations of NfL (standardized beta coefficient [β] = − 0.439, *p* < 0.001) and GFAP (β = − 0.321, *p* < 0.001).

**Conclusion:**

Patients with CKD had elevated plasma concentrations of NfL, p-Tau231 and GFAP compared with controls, and these biomarkers were inversely correlated with mGFR. Measured GFR was a significant, independent predictor of plasma concentrations of NfL and GFAP in patients with CKD. The mechanisms underlying this association need further investigation. Plasma levels of NfL and GFAP should be interpreted cautiously in patients with marked reductions in GFR.

**Supplementary Information:**

The online version contains supplementary material available at 10.1186/s12882-025-04130-2.

## Background

Patients with chronic kidney disease (CKD) have an increased risk of cerebrovascular disease and cognitive impairment [1, 2]. In addition to hypertension and cerebrovascular diseases, accumulation of uremic metabolites, anemia, and alterations in plasma concentrations of electrolytes and minerals may contribute to brain injury and cognitive dysfunctions [3–5]. Biomarkers of neurodegenerative diseases and brain injury can now be analysed in plasma and their concentrations have been shown to reflect levels in the cerebrospinal fluid (CSF) [6–8]. In patients with CKD, the interpretation of plasma concentrations of these biomarkers may be complicated if the kidneys contribute to their plasma clearance. Previous studies have found inverse correlations between estimated glomerular filtration rate (eGFR) and plasma concentrations of neurofilament light chain (NfL) [9, 10], tau [11, 12], and glial fibrillary acidic protein (GFAP) [13]. However, the mechanisms underlying these associations remain unclear.

In the present study we analysed plasma concentrations of three commonly used biomarkers of neurological disorders; NfL, tau phosphorylated at amino acid 231 (p-Tau231) and GFAP, in a well characterized cohort of patients with CKD stages 3 and 4 (eGFR 15–59 ml/min/1.73 m²) and examined their relationship to measured GFR (mGFR), urine albumin-to-creatinine ratio (U-ACR) and other abnormalities associated with severe CKD. As many confounding factors can influence plasma concentrations of creatinine and cystatin c, and thereby eGFR [14], it is essential to establish the relationship between plasma concentrations of neurological biomarkers and mGFR. Elevated U-ACR is a hallmark of glomerular barrier injury and an independent cardiorenal risk factor in patients with CKD [15]. Neurofilament light chain is a component of the axonal cytoskeleton in myelinated axons and a biomarker of neuronal axonal injury that increases in patients with neurodegenerative diseases and acute brain injury [16, 17]. Elevated levels of tau proteins are a consequence of axonal injury [18] and high peripheral levels of phosphorylated tau are characteristic for Alzheimer’s disease [19]. Glial fibrillary acidic protein is expressed by astrocytes [20], and a biomarker of astroglial activation [21].

The primary objective of this study was to investigate whether plasma levels of NfL, p-Tau231, and GFAP are elevated in patients with CKD stages 3 and 4, i.e. in a group of patients with marked reductions in GFR in which these biomarkers have not been comprehensively examined. Secondly, we aimed to identify independent predictors of plasma concentrations of these biomarkers with a particular focus on evaluating the role of mGFR. The secondary aim is important as patients with CKD stages 3 and 4 have multiple comorbidities and develop a range of cardiovascular and metabolic abnormalities that potentially could influence biomarkers of neurological disorders.

## Methods

### Subjects and protocol

This is a cross-sectional, observational, study on a cohort of adult (age ≥ 18 years) patients with CKD stages 3 and 4 recruited from the Nephrology outpatient clinic at Sahlgrenska University Hospital, Gothenburg, Sweden, between February 2009 and December 2011. Newly referred patients, or those with a planned follow-up within one month, were invited to participate, and 122 individuals were included. Patients needed to have an eGFR of 15–59 ml/min/1.73 m^2^ according to the Modification of Diet in Renal Disease (MDRD) formula for at least 3 months. Criteria for participation have been published previously [22]. As the aim of the present study was to examine biomarkers of neurological disorders in patients without manifest cerebral disease, 12 individuals were excluded because of a history of cerebrovascular disease (5 cerebral infarction, 1 cerebral bleeding, 2 subarachnoid bleeding and 4 transient ischemic attack). Patients were categorized as having atherosclerotic cardiovascular disease (ASCVD) if they had coronary artery disease, a diagnosis of peripheral artery disease, or a history of peripheral artery revascularization. Healthy, adult, controls were recruited through an advertisement in local newspapers, and 55 subjects with a similar age and gender distribution as patients with CKD, were included. Controls were not age-matched but there was no statistically significant difference between groups in age when all individuals were analysed (61.3 ± 11.7 vs 63.8 ± 10.8 years, in patients with CKD [*n* = 122] and controls [*n* = 55], respectively, *p* = 0.08). However, following exclusion of 12 patients with a history of cerebrovascular disease from the CKD cohort, patients with CKD were slightly, but statistically significantly, younger than healthy controls (Table 1). None of the study subjects had a diagnosis of dementia, cognitive impairment, or any other disease affecting the central nervous system. The study protocol did not include brain imaging to rule out subclinical cerebrovascular disease, nor tests to assess cognitive function. Smokers were defined as current or previous smokers, and non-smokers as individuals who had never smoked.

**Table 1:** General characteristics

	CKD (*n* = 110)	Healthy controls (*n* = 55)	*P*-value
Age, years	61 ± 12	64 ± 11	0.038
Men (%)	75 (68)	31 (56)	n.s.
Body weight, kg	83 ± 16	75 ± 14	0.002
BMI, kg/m^2^	27.0 ± 3.8	25.1 ± 3.3	0.001
Current or former smoker (%)	55 (50)	14 [26]	0.003
Hypertension (%)	96 (87)	0	<0.001
Diabetes (%)	31 (28)	0	<0.001
Atherosclerotic CV disease (%)	16 (15)	0	0.001
Ambulatory SBP, mmHg	124 ± 15	120 ± 12	n.s.
Ambulatory DBP, mmHg	73 ± 10	72 ± 7	n.s.
Carotid-femoral PWV, m/s	9.6 ± 2.7	8.7 ± 2.0	n.s.
mGFR, ml/min/1.73 m^2^	36 ± 15	85 ± 13	<0.001
S-creatinine, µmol/L	184 ± 72	76 ± 12	<0.001
S-cystatin c, mg/L	2.00 ± 0.66	0.92 ± 0.14	<0.001
U-ACR, mg/mmol	15 (3–73)	0 (0.0–0.4)	<0.001
B-Hemoglobin, g/L	127 ± 14	136 ± 11	<0.001
S-hsCRP, mg/L	3.25 ± 4.04	1.44 ± 2.03	<0.001
P-ApoB/ApoA1	0.75 ± 0.28	0.66 ± 0.17	0.030
S-phosphate, mmol/L	1.11 ± 0.23	1.02 ± 0.15	n.s.
S-PTH, pmol/L	10.9 ± 8.1	5.4 ± 1.7	<0.001
S-FGF-23, ng/L	185 ± 165	69 ± 19	<0.001
S-TnT, ng/L	13.1 (8.0–19.0)	5.7 (0.0–9.4)	<0.001
S-NTpro-BNP, ng/L	151 (78–285)	55 (33–98)	<0.001

Values are means ± SD or median (IQR). CKD, chronic kidney disease; BMI, body mass index; CV, cardiovascular; SBP, systolic blood pressure; DBP, diastolic blood pressure; mGFR, measured glomerular filtration rate, PWV, pulse-wave velocity; U-ACR, urine albumin-to-creatinine ratio; hsCRP, high-sensitivity c-reactive protein; PTH, parathyroid hormone; FGF-23, fibroblast growth-factor-23; S-TnT, serum troponin T; BNP, brain natriuretic peptide, and n.s., not significant. *P*-values are for the Mann-Whitney U test and for Chi-square with Fisher’s exact test

At study entry a detailed medical history was gathered, and the following analyses were performed: anthropometric measurements, urine and blood biochemistries, hemodynamic assessments, and plasma clearance of ^51^Cr-EDTA or iohexol to obtain mGFR. The Ethics Committee of the University of Gothenburg approved the study. The research was conducted in accordance with the Helsinki Declaration. All study subjects gave informed written consent to participate.

### Plasma analyses and measurement of GFR

Fasting blood samples were drawn in the morning from study subjects in a supine position and processed locally for routine analyses by standard laboratory methods as described [22]. For non-routine analyses, blood samples were immediately centrifuged at room temperature, and plasma aliquoted, and stored at − 70 °C until analysis. Urine albumin-to-creatinine ratio was analysed on urine collected for 24 h. Plasma clearance of ⁵¹Cr-EDTA, or iohexol, was measured according to clinical routines. Plasma clearance of these filtration markers has shown excellent agreement and is considered the gold standard techniques for measuring GFR [23]. Estimated GFR was calculated from plasma concentrations of creatinine and cystatin C using the CKD-EPI creatinine equation 2021 [24], and CKD-EPI cystatin C equation 2012 [25], respectively.

### Analyses of biomarkers of neurological disorders

Analyses were performed at the Neurochemistry Laboratory, Sahlgrenska University Hospital, Mölndal, Sweden. P-Tau231 was measured on EDTA-plasma using in-house immunoassays on the Single molecule array (Simoa) HD–X analyser (Quanterix) while NfL and GFAP were analysed using commercial assays from Quanterix (Billerica, MA, USA), following the instruction by the manufacturer, as earlier described [6, 26].

### Hemodynamic measurements

Ambulatory blood pressure (ABP) recordings for 24 h (Spacelabs Healthcare, Model 90217) and carotid-femoral pulse wave velocity (cfPWV) (SphygmoCor software version 8, AtCor Medical, Sydney, Australia) were measured as previously described [22].

### Statistics

Statistical analyses were performed using the SPSS software (IBM SPSS Statistics for Windows, Version 22.0. Armonk, NY, USA). Values are means ± standard deviations (SD) or medians with interquartile range (IQR) for continuous data, and numbers with proportions (%) for categorical variables. Statistical significance was set at the level of *p* < 0.05. Correlations between continuous data were analysed by determining Spearman´s rho. The Mann-Whitney U-test was used for comparing differences between groups in continuous data. Differences in frequencies were analysed using Fisher´s exact test. Multivariable linear regression analyses were performed to determine predictors of plasma concentrations of neurological biomarkers. Standardized beta coefficients (β) are presented for continuous variables and unstandardized beta coefficients for categorical variables. Based on our objectives stated in the “Background”, mGFR, U-ACR, B-hemoglobin, serum troponin T (S-TnT), and diabetes, were included as independent variables. Blood hemoglobin and S-TnT concentrations, reflecting renal anemia and subclinical coronary heart disease, showed statistically significant, linear relationships with neurological biomarkers in patients with CKD. Based on our bivariate correlation analyses, and results from previous studies [27, 28], age, BMI, gender and smoking status were also included as independent variables in regression models. Since the same independent variables were identified as probable predictors of the three different biomarkers, we used these variables in the three linear regression models. As plasma concentrations of NfL, p-Tau231 and GFAP were skewed we used log-transformed values as dependent variables in linear regression analyses. Approximation to a normal distribution of log-transformed data was confirmed by examining histogram and quantile-quantile plots. Linear relationships between continuous independent variables, and logarithmic plasma concentrations of biomarkers, were confirmed by inspecting scatterplots. Independent variables were examined for collinearity, and normal distribution of residuals was confirmed by inspecting the histogram and p–p plot.

## Results

## General characteristics

Patients with CKD were slightly younger, had higher body weight and BMI, and were more frequently smokers, compared to controls (Table 1). Twenty-eight percent of patients with CKD had diabetes (22 had type 2, and 9 had type 1, diabetes) and 15% had established ASCVD. There was no statistically significant difference between groups in 24 h ABPs. The use of antihypertensive drugs in patients with CKD was for angiotensin-converting enzyme (ACE) inhibitors or angiotensin receptor blockers (ARBs) 86%, diuretics 55%, calcium channel blockers 27%, beta-blockers 27%, and alpha-blockers 5%. Statins were used by 54% of patients with CKD and 34% were on antiplatelet therapy. The primary causes of CKD were glomerulonephritis (30%), diabetic kidney disease (23%), hypertension (11%), autosomal dominant polycystic kidney disease (9%), renovascular disease (3%) and other causes (24%). Patients with CKD had elevated serum levels of hsCRP, ApoB/ApoA1, PTH, FGF-23, TnT and NT-proBNP, while B-hemoglobin was significantly reduced, compared with controls. These abnormalities were anticipated and reflected marked reductions in mGFR in patients with CKD. There were no statistically significant differences between groups in serum phosphate, calcium, ferritin, or transferrin saturation (data not shown).

### Plasma concentrations of biomarkers of neurological disorders

**Fig. 1:**
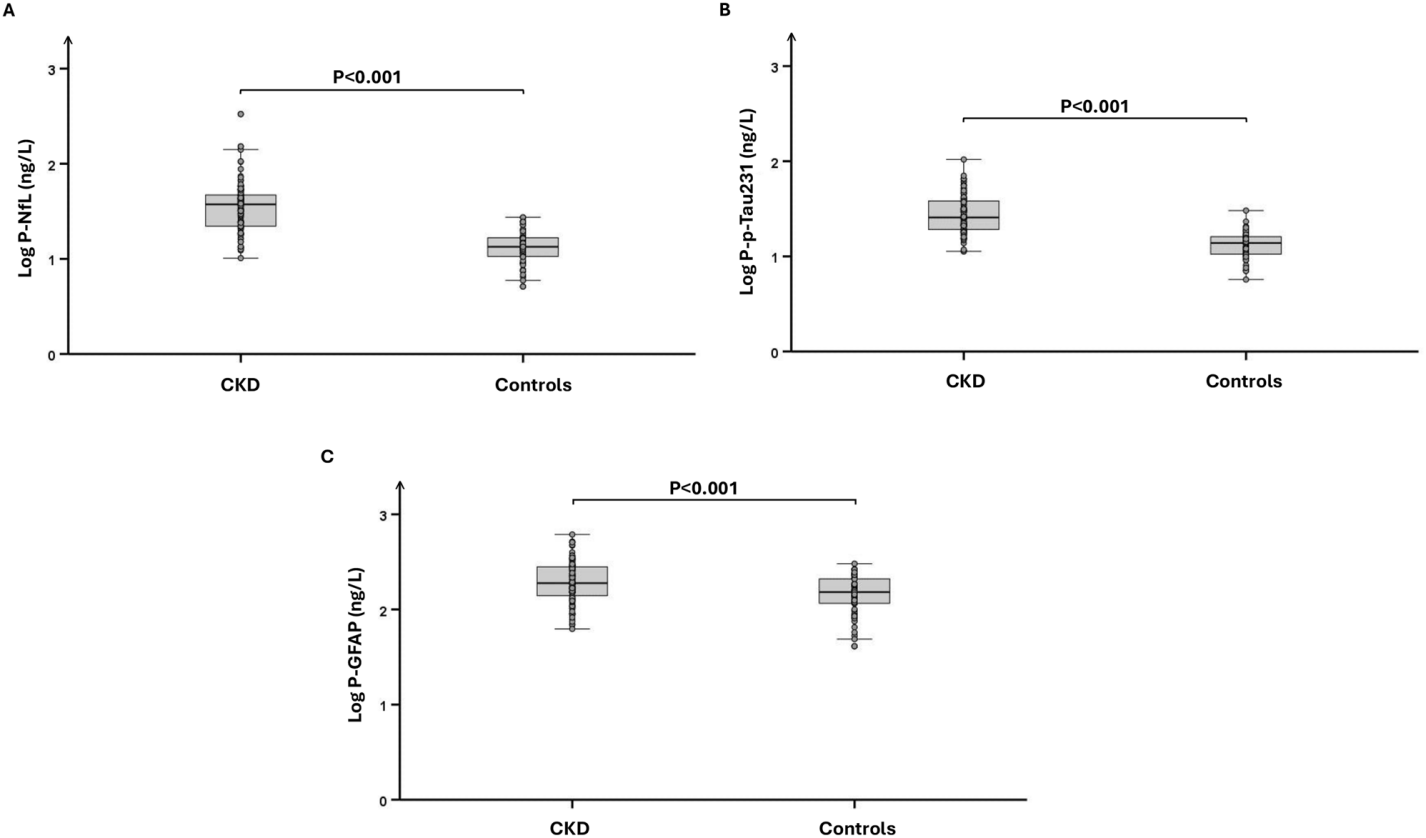
Plasma concentrations of biomarkers of neurological disorders. Box plots with individual data points showing log-transformed plasma concentrations of neurofilament light chain (NfL, **A**), phosphorylated Tau231 (p-Tau231, **B**) and glial fibrillary acidic protein (GFAP, **C**) in patients with chronic kidney disease (CKD, *n* = 110) and healthy controls (*n* = 55)

Plasma concentrations (median [IQR]) of NfL (37.5 [22.1–47.5] vs. 13.4 [10.5–16.7] ng/L, *p* < 0.001), p-Tau231 (25.7 [19.1–38.7] vs. 13.9 [10.5–16.3] ng/L, *p* < 0.001) and GFAP (190 [140–281] vs. 153 [116–211] ng/L, *p* < 0.001) were elevated in patients with CKD compared with controls. Log-transformed plasma concentrations of neurological biomarkers are shown in Fig. 1.

### Biomarkers of neurological disorders in patients with CKD

**Fig. 2:**
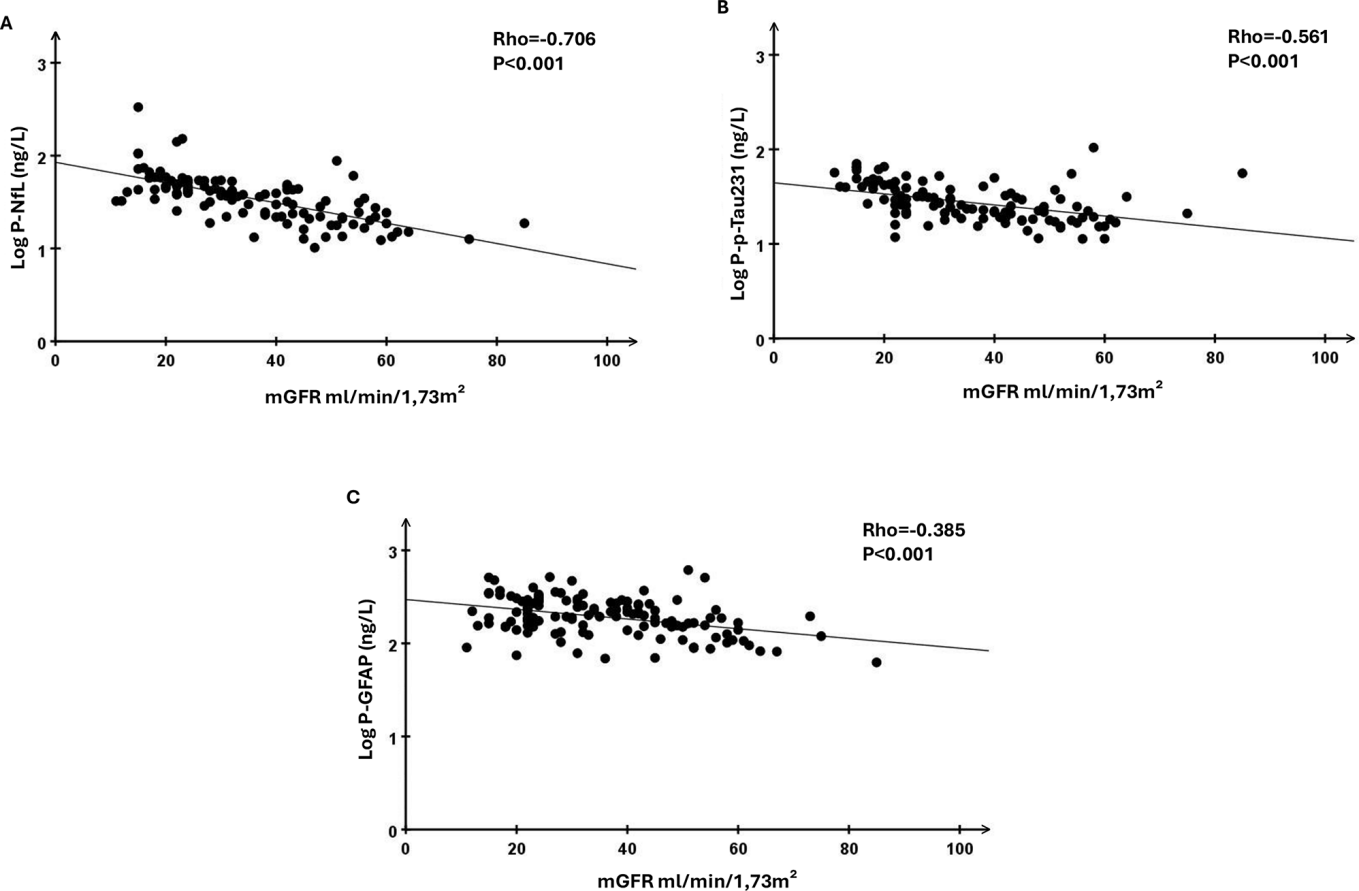
Correlations between mGFR and log-transformed plasma concentrations of biomarkers of neurological disorders in patients with CKD. Correlations between measured glomerular filtration rate (mGFR) and log-transformed plasma concentrations of neurofilament light chain (NfL, **A**), phosphorylated Tau231 (p-Tau231, **B**) and glial fibrillary acidic protein (GFAP, **C**) in patients with chronic kidney disease (CKD, *n* = 110). Linear regression lines are depicted

Spearman’s correlation coefficients between clinical variables and biomarkers are shown in Table 2. Measured GFR, and eGFR based on creatinine or cystatin C (data not shown), all showed statistically significant, negative, correlations to plasma levels of NfL, p-Tau231 and GFAP (Fig. 2). Age displayed a statistically significant, positive, correlation to plasma concentrations of NfL and GFAP, but was not significantly correlated to p-Tau231. Plasma concentrations of neurological biomarkers also showed statistically significant correlations to each other. Neither hsCRP, nor plasma albumin, showed statistically significant correlations to any of the neurological biomarkers.

**Table 2:** Spearman’s correlation coefficient between clinical variables and plasma concentrations of neurological biomarkers in patients with chronic kidney disease

	P-NfL, ng/L	P-p-Tau231, ng/L	P-GFAP, ng/L
Age, years	0.357***	−0.01	0.554***
BMI, kg/m^2^	−0.051	−0.072	−0.137
B-Hemoglobin, g/L	−0.304**	−0.458***	−0.202*
mGFR, ml/min/1.73 m^2^	−0.706***	−0.561***	−0.385***
eGFR_creatinine_, ml/min/1.73 m^2^	−0.656***	−0.511***	−0.322**
U-ACR, mg/mmol	0.238*	0.229*	−0.031
Carotid-femoral PWV, m/s	0.354***	0.136	0.395***
S-TnT, ng/L	0.613***	0.412***	0.395***
P-NfL, ng/L	–	0.553***	0.642***
P-p-Tau231, ng/L	0.533***	–	0.284**

BMI, body mass index; mGFR, measured glomerular filtration rate; eGFR_creatinine_, estimated GFR based on plasma-creatinine; U-ACR, urine albumin-to-creatinine ratio; PWV, pulse-wave velocity; S-TnT, serum troponin T; NfL, neurofilament light chain; p-Tau231, phosphorylated Tau231; and GFAP, Glial fibrillary acidic protein. * denotes *p* < 0.05, ** denotes *p* < 0.01, *** denotes *p* < 0.001. *N* = 110

Patients with CKD and diabetes had significantly elevated plasma concentrations of NfL (44.8 [36.4–58.6] vs. 32.4 [20.1–43.1] ng/L, *p* < 0.001) and p-Tau231 (31.7 [24.0–46.6] vs. 23.4 [18.6–32.5] ng/L, *p* = 0.018), but not of GFAP (207 [151–315] vs. 187 [130–257] ng/L, *p* = 0.10), compared with patients without diabetes. There were no statistically significant differences in plasma levels of neurological biomarkers between males and females, or between patients with or without ASCVD or a smoking history.

## Predictors of biomarkers of neurological disorders in patients with CKD

Multivariable linear regression analyses including age, BMI, mGFR, U-ACR, B-hemoglobin, S-TnT, gender, diabetes, and smoking as independent variables were performed to predict log-transformed plasma concentrations of NfL, p-Tau231 and GFAP. Variance inflation factors did not exceed 2.0 for any independent variable, in any of the regression models, indicating minimal collinearity.

Independent, significant, predictors of log-transformed plasma NfL were mGFR, age and S-TnT (Table 3). The model had an R^2^ of 0.622 with an adjusted R^2^ of 0.587.

**Table 3:** Beta coefficients from multivariable linear regression analyses in patients with chronic kidney disease

	log p-NfL (ng/L)	log p–p-Tau231 (ng/L)	log p-GFAP (ng/L)
Age, years	0.264**	−0.233**	0.616***
BMI, kg/m^2^	−0.079	0.030	−0.189*
mGFR, ml/min/1.73 m^2^	−0.439***	−0.108	−0.321***
U-ACR, g/mol	0.145	0.017	0.015
B-Hemoglobin, g/L	−0.105	−0.310**	−0.032
S-TnT, ng/L	0.244**	0.548***	−0.013
Male gender	−0.032	−0.001	−0.045
Smoking	−0.010	0.001	−0.078**
Diabetes	0.058	0.023	0.012

Dependent variables are log-transformed plasma concentrations of neurological biomarkers. Standardized beta coefficients are presented for continuous variables and unstandardized beta coefficients for categorical variables. BMI, body mass index; mGFR, measured glomerular filtration rate; eGFR_creatinine_, estimated GFR based on plasma-creatinine; U-ACR, urine albumin-to-creatinine ratio; S-TnT, serum troponin T; NfL, neurofilament light chain; p-Tau231, phosphorylated Tau231; and GFAP, Glial fibrillary acidic protein. * denotes *p* < 0.05, ** denotes *p* < 0.01, *** denotes *p* < 0.001. *N* = 110

Independent, significant, predictors of log-transformed plasma p-Tau231 were S-TnT, B-hemoglobin and age (Table 3). Notably, increased age was associated with reduced plasma concentrations of p-Tau231. The model had an R^2^ of 0.497 with an adjusted R^2^ of 0.45.

Independent, significant, predictors of log-transformed plasma GFAP were age, mGFR, BMI, and smoking status with lower plasma concentrations of GFAP in patients with a smoking history (Table 3). The model had an R^2^ of 0.555, with an adjusted R^2^ of 0.514.

### Biomarkers of neurological disorders in all study subjects (CKD + controls)

As we observed statistically significant correlations between mGFR and plasma concentrations of NfL and GFAP also in healthy controls (see Supplemental Table 1), we performed correlation and regression analyses that included all study subjects by pooling patients with CKD and healthy controls (*n* = 165). Spearman’s correlation coefficients between clinical variables and neurological biomarkers are shown in Table 4. Measured GFR, as well as eGFR based on creatinine or cystatin C (data not shown), all showed statistically significant, negative, correlations with plasma levels of NfL, p-Tau231 and GFAP.

**Table 4:** Spearman’s correlation coefficient between clinical variables and plasma concentrations of neurological biomarkers in all study subjects (CKD + controls)

	P-NfL, ng/L	P-p-Tau231, ng/L	P-GFAP, ng/L
Age, years	0.180*	−0.132	0.433***
BMI, kg/m^2^	0.069	0.098	−0.102
B-Hemoglobin, g/L	−0.390***	−0.442***	−0.260**
mGFR, ml/min/1.73 m^2^	−0.846***	−0.735***	−0.449***
eGFR_creatinine_, ml/min/1.73 m^2^	−0.822***	−0.730***	−0.402***
U-ACR, mg/mmol	0.588***	0.587***	0.186*
Carotid-femoral PWV, m/s	0.323***	0.147	0.376***
S-TnT, ng/L	0.640***	0.539***	0.378***
P-NfL, ng/L	–	0.714***	0.605***
P-p-Tau231, ng/L	0.714***	–	0.336***

BMI, body mass index; mGFR, measured glomerular filtration rate; eGFR_creatinine_, estimated GFR based on plasma-creatinine; U-ACR, urine albumin-to-creatinine ratio; PWV, pulse-wave velocity; S-TnT, serum troponin T; NfL, neurofilament light chain; p-Tau231, phosphorylated Tau231; and GFAP, Glial fibrillary acidic protein. * denotes *p* < 0.05, ** denotes *p* < 0.01, *** denotes *p* < 0.001. *N* = 165

Smokers had significantly elevated plasma concentrations of NfL compared with non-smokers (31.7 [17.8–43.9] vs. 22.9 [13.2–40.7] ng/L, *p* = 0.027) and plasma p-Tau231 levels were higher in males vs. females (26.8 [15.5–32.0] vs. 21.5 [12.8–24.9] ng/L, *p* = 0.019).

### Predictors of biomarkers of neurological disorders in all study subjects (CKD + controls)

Multivariable linear regression analyses to predict log-transformed plasma concentrations of NfL, p-Tau231 and GFAP were performed using the same nine independent variables that were applied when the CKD-group was analysed separately (*see above*). Variance inflation factors did not exceed 2.0 for any independent variable, in any regression models, indicating minimal collinearity.

Independent, significant, predictors of log-transformed plasma NfL were mGFR, age, S-TnT, BMI, U-ACR and diabetes (Table 5). Elevated BMI was associated with reduced plasma NfL concentrations. The regression model had an R^2^ of 0.774, with an adjusted R^2^ of 0.760.

**Table 5:** Beta coefficients from multivariable linear regression analyses in all study subjects (CKD + controls)

	log p-NfL (ng/L)	log p-p-Tau231 (ng/L)	log p-GFAP (ng/L)
Age, years	0.196***	−0.214***	0.494***
BMI, kg/m^2^	−0.119**	−0.005	−0.180**
mGFR, ml/min/1.73 m^2^	−0.658***	−0.357***	−0.446***
U-ACR, g/mol	0.112*	−0.005	−0.003
B-Hemoglobin, g/L	−0.034	−0.240***	−0.060
S-TnT, ng/L	0.172**	0.380***	0.043
Male gender	−0.014	0.044	−0.054
Smoking	−0.004	0.013	−0.053*
Diabetes	0.074*	0.041	0.021

Dependent variables are log-transformed plasma concentrations of neurological biomarkers. Standardized beta coefficients are presented for continuous variables and unstandardized beta coefficients for categorical variables. BMI, body mass index; mGFR, measured glomerular filtration rate; eGFR_creatinine_, estimated GFR based on plasma-creatinine; U-ACR, urine albumin-to-creatinine ratio; S-TnT, serum troponin T; NfL, neurofilament light chain; p-Tau231, phosphorylated Tau231; and GFAP, Glial fibrillary acidic protein. * denotes *p* < 0.05, ** denotes *p* < 0.01, *** denotes *p* < 0.001. *N* = 165

Independent, significant, predictors of log-transformed plasma p-Tau231 were S-TnT, mGFR, B-hemoglobin, and age (Table 5). Increased age was associated with reduced plasma concentrations of p-Tau231. The model had an R^2^ of 0.631, with an adjusted R^2^ of 0.609.

Independent, significant, predictors of log-transformed plasma GFAP were age, mGFR, BMI and smoking history (Table 5). Both increased BMI and smoking history were associated with reduced plasma concentrations of GFAP. The model had an R^2^ of 0.507, with an adjusted R^2^ of 0.478.

## Discussion

The main finding of the present study was that patients with CKD stages 3 and 4, without a diagnosis of cerebrovascular disease or dementia, had elevated plasma concentrations of NfL, p-Tau231 and GFAP compared with controls with normal mGFR. In addition, mGFR was inversely correlated to plasma levels of neurological biomarkers, and independently predicted plasma concentrations of NfL and GFAP in patients with CKD using multivariable regression models. In the pooled cohort, including both patients with CKD and healthy controls, mGFR also independently predicted plasma concentrations of p-Tau231. Furthermore, we found an independent, positive association between S-TnT and plasma concentrations of NfL and p-Tau231.

The mechanisms underlying the inverse correlation between mGFR and plasma concentrations of neurological biomarkers remain to be determined. In theory, these associations could be explained either by reduced renal elimination of the biomarkers or by increased release from the nervous system due to injury. Renal elimination of plasma proteins occurs through glomerular filtration which is a highly size-selective process. Hence, the concentration of albumin (molecular weight of approximately 69 kDa) in primary urine is estimated to be approximately 0.01% of that in plasma, and urinary albumin excretion is normally < 30 mg per day [29, 30]. Molecular weights of NfL (68 kDa), p-Tau231 (48–67 kDa) and GFAP (55 kDa) are close to that of albumin indicating that renal elimination of these proteins is low and should only have a minor influence on plasma concentrations. However, we cannot rule out the possibility that the immunoassays used in the present study may have detected smaller peptide fragments of the biomarkers that could be cleared from plasma through glomerular filtration. This possibility needs to be investigated further. In accord with our results, previous studies have shown that eGFR predicts plasma concentrations of NfL independently of age [9, 31]. We confirm and extend these findings by demonstrating that this association was highly significant also with mGFR analysed using state-of-the-art methodology with exogenous filtration markers. In addition, the β coefficient for mGFR was much higher than that for age in the regression model predicting plasma NfL concentrations in patients with CKD, suggesting that mGFR was a stronger predictor than age in this cohort.

The association between U-ACR and plasma NfL has been less investigated. Elevated U-ACR is a hallmark of glomerular diseases but may also reflect generalized endothelial dysfunction, for instance in patients with diabetes or hypertension [15]. In the pooled cohort we found an independent, positive association between U-ACR and plasma concentrations of NfL. Similarly, diabetes was independently associated with elevated plasma levels of NfL. Notably, these associations were independent of mGFR and might reflect a connection between generalized endothelial dysfunction and elevated plasma NfL. Interestingly, plasma NfL predicted incident stroke in a cohort of patients with type 2 diabetes from the ACCORD (Action to Control Cardiovascular Risk in Diabetes) trial, and in this study, CKD was strongly associated with increased NfL concentrations [31]. It is well established that CKD is associated with accelerated vascular disease and that cardiovascular disease is the major cause of death in this population [32]. Several studies have shown that S-TnT is increased in clinically stable patients with CKD and associated with increased cardiovascular risk [33] and subclinical myocardial dysfunctions [34]. Interestingly, we found an independent, positive association between S-TnT and concentrations of both NfL and p-Tau231 in patients with CKD in the present study. We speculate that CKD, through multiple mechanisms, causes microvascular abnormalities that in parallel may lead to subclinical injuries in the brain and myocardium.

In a subgroup of a large population-based cohort of 70-year-old individuals, Dittrich et al. [10] found that CKD (eGFR < 60 ml/min/1.73 m^2^) was associated with elevated plasma NfL but not with increased NfL concentrations in the CSF. Similarly, Pichet Binette et al. [35] investigated the association between plasma creatinine levels and neurological biomarkers measured in plasma and CSF of two large Swedish cohorts with older participants. Increased plasma creatinine was associated with elevated plasma concentrations of NfL and GFAP, and to a lesser extent with phosphorylated tau, resembling findings in the present study. However, adjustment for plasma creatinine had only minor effects in models predicting either corresponding biomarker levels in the CSF, or future development of dementia. In a large population-based cohort study with 17-years of follow-up, Stocker et al. [12] found that plasma concentrations of NfL and p-tau181 were elevated in individuals with CKD but not associated with an increased dementia risk. Taken together, these findings suggest that reduced GFR mainly influences plasma concentrations of the abovementioned biomarkers by reducing their plasma clearance and is likely a confounding factor when assessing CNS pathology.

Our study has obvious strengths and limitations. Importantly, we present data on mGFR and can conclude that results were like those derived from eGFR. This is important as several confounding factors can affect plasma concentrations of creatinine and cystatin C and hence eGFR [14]. In addition, we present data on U-ACR which enabled us to investigate the separate influence of albuminuria and mGFR on plasma levels of neurological biomarkers. Furthermore, in contrast to most previous studies, we included patients with a low eGFR down to 15 ml/min/1.73 m^2^. As for limitations, our cohort was relatively small, and we did not collect CSF samples which prevented us from relating biomarker concentrations in plasma to those in the CSF. In addition, study participants did not undergo per protocol brain imaging or cognitive function tests. We can therefore not rule out subclinical brain injuries in our study subjects. Moreover, we were unable to examine associations between plasma biomarker concentrations and cognitive function.

## Conclusion

Patients with CKD stages 3 and 4, without cerebrovascular disease, had elevated plasma concentrations of NfL, p-Tau231 and GFAP compared with healthy controls, and mGFR was inversely correlated to plasma levels of these biomarkers. In addition, mGFR was an independent predictor of plasma concentrations of NfL and GFAP in patients with CKD. The mechanisms underlying these associations need to be investigated further. Our results suggest that plasma concentrations of NfL, and GFAP should be interpreted cautiously in patients with marked reduction in GFR.

## Electronic supplementary material

Below is the link to the electronic supplementary material.

Supplementary Material 1

## Data Availability

The data used and analyzed in this study are available from the corresponding author on reasonable request.

## Abbreviations:

CKD: Chronic kidney disease
NfL: Neurofilament light chain
GFAP: Glial fibrillary acidic protein
P-Tau231: Phosphorylated Tau231
SIMOA: Single molecule array
CSF: Cerebrospinal fluid
GFR: Glomerular filtration rate
eGFR: Estimated glomerular filtration rate
mGFR: Measured glomerular filtration rate
MDRD: Modification of Diet in Renal Disease
BMI: Body mass index
ASCVD: Atherosclerotic cardiovascular disease
U-ACR: Urine albumin-to-creatinine ratio
ACE: Angiotensin-converting enzyme
ARB: Angiotensin receptor blocker
ABP: Ambulatory blood pressure
cfPWV: Carotid-femoral pulse wave velocity
