# Supplementary material for: Plasma concentrations of neurofilament light, p-Tau231 and glial fibrillary acidic protein are elevated in patients with chronic kidney disease and correlate with measured glomerular filtration rate
Source: BMC Nephrol. 2025 May 9;26:231. doi: 10.1186/s12882-025-04130-2 (PMC12065258; doi:10.1186/s12882-025-04130-2)
Supplement: Supplementary file 1 — Supplementary Material 1 [file 12882_2025_4130_MOESM1_ESM.docx]

**Supplemental table 1. Spearman’s correlation coefficient between clinical variables and plasma concentrations of neurological biomarkers in healthy controls.**

|  | P-NfL, ng/L | P-p-Tau231, ng/L | P-GFAP, ng/L |
| --- | --- | --- | --- |
| Age, years | 0.575*** | 0.051 | 0.431** |
| BMI, kg/m^2^ | -0.392** | -0.217 | -0.288* |
| B-Hemoglobin, g/L | 0.011 | -0.085 | -0.264 |
| mGFR, ml/min/1.73m^2^ | -0.610*** | 0.104 | -0.400** |
| eGFR_creatinine_, ml/min/1.73m^2^ | -0.534*** | 0.028 | -0.288* |
| U-ACR, mg/mmol | 0.209 | 0.038 | 0.111 |
| Carotid-femoral PWV, m/s | 0.335* | -0.015 | 0.299* |
| S-TnT, ng/L | 0.144 | 0.299* | 0.013 |
| P-NfL, ng/L | - | 0.168 | 0.475*** |
| P-p-Tau231, ng/L | - | - | 0.064 |

BMI, body mass index; mGFR, measured glomerular filtration rate; eGFR_creatinine_, estimated GFR based on plasma-creatinine; U-ACR, urine albumin-to-creatinine ratio; PWV, pulse-wave velocity; S-TnT, serum troponin T; NfL, neurofilament light chain; p-Tau231, phosphorylated Tau231; and GFAP, glial fibrillary acid protein. * denotes p<0.05, ** denotes p<0.01, *** denotes p<0.001. N=55.
